# Selenium and Selenoproteins in Neutrophil Functions

**DOI:** 10.1007/s12011-025-04822-8

**Published:** 2025-09-18

**Authors:** Tai-Jung Lee, K. Sandeep Prabhu

**Affiliations:** Department of Veterinary & Biomedical Sciences, Center for Molecular Immunology and Infectious Disease, and Center for Molecular Toxicology and Carcinogenesis, The Pennsylvania State University, University Park, PA 16802 USA

**Keywords:** Efferocytosis, Inflammation, NETs, Redox

## Abstract

Neutrophils are innate immune cells, whose activation leads to extensive production of reactive oxygen species (ROS) through the activation of NADPH oxidases (NOXs). ROS plays a pivotal role in modulating neutrophil functions, including phagocytosis, migration, release of neutrophil extracellular traps (NETs), activation of proinflammatory signaling pathways, and apoptosis. Selenium is an essential micronutrient antioxidant that exhibits biological functions through its translational incorporation as the 21st amino acid selenocysteine. With their diverse enzymatic activities, selenium and selenoproteins partake in the modulation of immune cell activities through regulating multiple cellular functions, including redox balance and antioxidant defense. Given the critical role of ROS in neutrophil function, selenium and selenoproteins are likely to modulate neutrophil activities through regulating both redox-dependent and -independent signaling pathways. Here, we review the current understanding of the role of selenium and selenoproteins in regulating neutrophil functions.

Neutrophils are innate immune cells that are among the first responders recruited to the site of inflammation, playing a pivotal role not only in the initiation of an inflammatory response, but also its timely resolution. Neutrophils are professional phagocytes, particularly toward bacteria that culminate in intracellular or extracellular killing [1]. Neutrophils express various receptors such as Fc receptors, pathogen-recognition receptors (PRRs), and chemokine/cytokine receptors, facilitating their recognition of danger- and pathogen-associated molecular patterns (DAMPs and PAMPs), and communication with surrounding cell types. These interactions lead to the activation of neutrophil inflammatory signaling pathways to execute the effector functions, including migration, phagocytosis, degranulation, and the release of neutrophil extracellular traps (NETs) [1]. Receptor-mediated signaling cascades also amplify proinflammatory responses through NFκB-mediating activation of inflammasome, facilitating the secretion of IL-1β and IL-18 [1]. Moreover, activation of proinflammatory signaling pathways also induces the production of immunomodulatory lipid mediators, including those from eicosanoid metabolism, through upregulation of the expression of eicosanoid enzymes such as cyclooxygenases (COXs) and lipoxygenases (LOXs) and their respective bioactive lipid products, prostaglandins (PG), thromboxanes, and leukotriene B4 (LTB_4_) [2]. These bioactive lipids act in an autocrine and paracrine manner to modulate the recruitment and activation of neutrophils, as well as other proinflammatory immune cells, together facilitating the eradication of inflammatory stimuli thereby setting the stage for resolution of inflammation [2].

The cytotoxic functions make neutrophils efficient “killers” against invading pathogens; however, these actions also cause collateral tissue damage [3]. Degranulation-dependent release of microbicidal proteins combined with the various reactive oxygen species (ROS) leads to host tissue damage, while the release of NETs serves as DAMPs, which further activate the immune cells in the immune microenvironment, leading to unresolved inflammation [3]. Thus, the dysregulation of neutrophil functions contributes to the pathogenesis of various inflammatory diseases, including inflammatory bowel disease (IBD) and autoimmune diseases [3].

ROS play a critical role in regulating the function of neutrophils [4]. Unlike other cells where the ROS are usually a byproduct of mitochondrial oxidative phosphorylation [5], neutrophils produce ROS through NADPH oxidases (NOXs), particularly the NOX2 complex [4]. The activation of receptor signaling pathways promotes the active assembly of NOX complex, where the cytosolic subunits p40^phox^, p47^phox^, p67^phox^, and small GTPase Rac are transported to and assembled with membrane resident subunits gp91^phox^ (NOX2) and p22^phox^, leading to the generation of superoxide anions (O_2_•^-^) from O_2_ at the expense of reducing equivalents (NADPH) [4]. The O_2_•^-^ is further dismutated to H_2_O_2_, which can act as a substrate for myeloperoxidase (MPO) for the generation of hypochlorous acid (HOCl) to facilitate microbial killing in the phagosome [4]. In addition to its bactericidal activity, ROS also function as signaling messengers to modulate various neutrophilic functions, including the formation of NETs by activating and promoting nuclear translocation of neutrophil elastases (NE), MPO, and peptidylarginine deiminase 4 (PAD4) [4, 6], as well as the activation of proinflammatory signaling pathways through NFκB activation [7], which altogether contribute to the regulation of neutrophil cell death including apoptosis and ferroptosis [8]. Therefore, the regulation of neutrophil redox homeostasis is essential for controlling neutrophil inflammatory activity.

Selenium is an essential micronutrient that exhibits its biological role in the form of the 21st amino acid selenocysteine (Sec) [9]. Due to the low redox potential of Sec, the Sec-containing proteins are more efficient as redox gatekeepers in protecting cells from oxidative damage [10]. Organic or inorganic forms of selenium acquired through diet are absorbed in the intestine and transported to the liver by very low-density lipoprotein (VLDL) and low-density lipoprotein (LPL) [11]. Selenium is then converted to selenophosphate via selenophosphate synthetase 2 (SPS2), which is used to charge a specific tRNA^[Ser]Sec^ encoded by tRNA-SeC (anticodon TCA) 1-1 (*TRU-TCA1-1; TRSP*). This charged tRNA enables the co-translational incorporation of Sec through the UGA codon in the presence of Sec Insertion Sequence (SECIS), a conserved stem-loop structure of selenoproteins [9, 11]. Liver and other tissues secrete selenoprotein P (SELENOP), a selenoprotein with 10 Sec residues, which can be acquired by cells through receptor-mediated endocytosis, following which it is digested to release Sec for synthesis of other selenoproteins [12]. There are 24 selenoproteins in mice and 25 in human, and can be generally categorized based on their functions, including glutathione peroxidases (GPX), which mediate glutathione-dependent redox defense, thioredoxin reductase (TXNRD) that catalyzes the reduction of oxidation-induced disulfide formation in thioredoxin and other proteins, and deiodinases (DIO), which regulate thyroid hormone activation [9, 12]. In addition, some of the selenoproteins whose functions have remained largely unknown are now being gradually elucidated in the context of various pathophysiological conditions such as Alzheimer’s diseases [13], muscle regeneration [14, 15], chronic inflammatory diseases [16, 17], and cancers [9]. Molecular functionality of various selenoproteins is also being deciphered, such as SELENON and SELENOK in regulating Ca^2+^ homeostasis [15, 18], SELENOI in catalyzing phospholipid synthesis [19], and SELENOW in regulating cell cycle progression and proliferation signaling pathways [20]. Due to the oxidative stress encountered by immune cells, selenoproteins have been found to be involved in regulating the functions of T cells, B cells, NK cells, and macrophages [9, 16]. Of particular interest, the significant role of ROS during neutrophil activation suggests the potential involvement of selenoproteins as important redox gatekeepers in regulating the function of neutrophils. Here, we review the current literature on the role of selenium and selenoproteins in the regulation of neutrophil functions.

## Selenium and Neutrophil ROS Production

Various studies carried Out in the 1980s and 1990s investigated the functions of neutrophil during selenium deficiency in cattle, and later were validated in rodents, where the isolated neutrophils showed reduced efficiency in intracellular killing of yeast and bacteria [21, 22]. The reduced intracellular killing of pathogens was likely a result of decreased ROS production [21], which is required for the lysis of engulfed pathogens in the lysosome. Interestingly, the ability for ROS production in neutrophils as a function of selenium status seems to vary between studies. Despite most of the animal studies that showed selenium deficiency downregulated neutrophil ROS production [21–23], human studies showed that low serum selenium level was associated with increased ROS production by neutrophils, and selenium supplementation was associated with reduced intracellular killing ability of human neutrophils [24]. In addition, treatment of selenium-containing nanoparticle (SeNP) significantly downregulated neutrophil ROS production upon N-formyl-methionyl-leucyl-phenylalanine (fMLP) and zymosan stimulation [25]. Our studies with selenoprotein-depleted neutrophils also showed an increased ROS production against LPS [22] and *Citrobacter rodentium* (*C. rodentium*) stimulation [26]. The discrepancy in the ROS generating ability of selenium suggests that selenium and selenoproteins might be involved in the fine-tuning of the ROS production in neutrophils.

## Selenium and Phagocytosis

The effect of selenium on the phagocytic ability in neutrophils also appeared to be varied between studies. Neutrophils isolated from selenium-deficient or supplemented cattle showed no difference in phagocytic ability toward *Candida albicans* or *Escherichia coli* [27, 28]. On the contrary, selenium was found to promote neutrophil phagocytosis in humans [29], swine [23], and sheep [22], and selenoprotein-depleted neutrophils also showed reduced phagocytosis ability toward bacteria [22]. Phagocytosis is a complex process regulated by various signaling pathways, beginning with the engagement of recognition receptors such as Fcγ receptors, complement receptors, or PRRs, which activate downstream phosphatidylinositol 3-kinase (PI3K) and phospholipase C (PLC) signaling cascade and promote the activation of small GTPase Rac or Rho that in turn mediate cytoskeletal rearrangement required for the formation of phagosomes and active assembly of NOX2 [4]. PLC- and PI3K-mediated Ca^2+^ signaling pathways are also involved in phagosome maturation and fusion with secretory granules [4]. Selenoproteins, including selenoprotein M (SELENOM), SELENON, SELENOT, SELENOK, methionine sulfoxide reductase B1 (MSRB1), and SELENOW, were reported to be involved in multiple signaling events such as cytoskeletal regulation [30], Ca^2+^ signaling [18], and Rac1 activation [14], which serve as critical events essential for phagocytosis. Considering the hierarchy in the expression of selenoproteins under varying selenium and pathophysiological conditions [31], it likely contributes to the discrepancy in the phagocytic ability that would benefit from additional studies to investigate the detailed mechanism underlying the effect of selenium and selenoproteins in modulating neutrophil phagocytosis.

## Selenium and Neutrophil Migration

Selenium levels were found to modulate neutrophil chemotactic activity. Selenium supplementation of the culture media rescued the chemotaxis ability toward fMLP of bovine neutrophils on a selenium-deficient diet [32]. Administration of selenium nanoparticles (SeNP) increased neutrophil adhesion and expression of integrin and selectin and enhanced neutrophil migration to the peritoneum following zymosan-induced peritonitis [25]. ROS have been found to mediate neutrophil migration. Neutrophils isolated from chronic granulomatous disease (CGD) patients (with a defective NADPH oxidase) or healthy neutrophils treated with NADPH oxidase inhibitors exhibited impaired migration and chemotaxis toward fMLP [26]. In addition, ROS production is associated with neutrophil rolling and expression of adhesion molecule L-selectin and CD11b [33]. Interestingly, epithelial cells cultured without selenium showed increased neutrophil adhesion molecule expression [34]. Our laboratory demonstrated that knockout of the neutrophil-specific selenoproteome, achieved by deleting the Sec tRNA[Ser]Sec (*Trsp*) in S100A8-expressing granulocytes (Trsp^fl/fl^S100A8^Cre^), led to a marked increase in neutrophil accumulation in the colons of Trsp^fl/fl^S100A8^Cre^ mice during *C. rodentium* infection [26]. Selenoprotein-deficient neutrophils exhibited a significant increase in the expression of adhesion molecule integrin subunit alpha M (ITGAM; CD11b) and chemokine receptor C-X3-C motif chemokine receptor 1 (CX3CR1), along with enhanced chemotaxis toward fMLP and chemokine C-X3-C motif chemokine Ligand 1 (CX3CL1) [26]. In addition, stimulation with LPS increased arachidonate 12-lipoxygenase (ALOX12) expression in selenoprotein-deficient neutrophils [22]. ALOX12 catalyzes the production of 12(*S*)-hydroxyeicosatetraenonic acid (12-HETE) from arachidonic acid, which has been found to elicit neutrophil proinflammatory functions, including degranulation and migration [22]. Furthermore, selenium deficiency also downregulated proinflammatory arachidonic acid downstream lipid mediator leukotriene B4 (LTB_4_) production in caprine neutrophils, another arachidonic acid–derived ALOX product that promotes neutrophil chemotaxis [35]. These results suggest that selenium, through selenoproteins, regulates ROS production in neutrophils to likely modulate lipid mediator production and adhesion molecular expression, and therefore, modulate neutrophil chemotaxis and migration.

## Selenium and NETs

ROS regulate the formation of NETs, a net-like structure formed by decondensed chromatin decorated with cytoplasmic and granule proteins MPO, neutrophil elastase, and proteases, which facilitates the trapping and opsonization of pathogen when released extracellularly [1]. Selenium is found to modulate NETs forming ability of neutrophils. Low level of selenium (< 0.01 mg/l) in culture media induced NETs formation of bovine neutrophils under naïve conditions; however, the effect was reversed when neutrophils were cultured with higher concentrations of selenium (0.08 mg/l and 2 mg/l; as selenite) [32]. Similar results were observed in mice injected with SeNP [25]. On the contrary, selenium supplementation appeared to prevent NETs formation during inflammation. Selenium supplementation and SeNP treatment inhibited the NETs-forming ability in neutrophils isolated from atherosclerosis patients [36], and in mice with rheumatoid arthritis (RA) [37], respectively. Zymosan-induced NETs formation was also inhibited in neutrophils isolated from mice treated with SeNP in a dose-dependent manner [25]. On the other hand, a few studies investigating the proinflammatory effects of heavy metals and toxins showed that selenium supplementation could enhance neutrophil proinflammatory function via upregulated NETs formation [38, 39]. In our own studies using murine models, neutrophils lacking the selenoproteome displayed impaired NETs release in contrast to wild-type (WT) neutrophils, where co-culture with *C. rodentium* failed to induce an increase in NET formation, perhaps due to an increased baseline production of NETs [22]. Given that ROS are important activation signals for NET formation, promoting the activation of NE and PAD4 accelerates the decondensation of chromatin and subsequent rupture of the nuclear membrane [6]. Additionally, selenium deficiency increased thromboxane A_2_ synthesis [40], which regulates inflammatory functions of neutrophils including degranulation of NE [41] and NET formation [42]. Together, these findings suggest that ROS accumulation in neutrophils and their capacity for NET formation are influenced by selenium status, which in turn shapes the pathways that govern inflammation resolution.

## Selenium and Inflammatory Cytokine Production

Selenium is found to affect proinflammatory cytokine production in neutrophils. Upregulated NFκB signaling pathway and production of IL-1β and TNFα in neutrophils from both naïve and disease conditions were observed during selenium deficiency [23, 43]. Trsp^fl/fl^S100A8^Cre^ neutrophils also showed increased NFκB activation, with increased IL-1β and TNFα expression [22]. Furthermore, SeNP treatment inhibited proinflammatory cytokines production in RA [37]. In addition to the activation of NFκB pathway, ROS was reported to trigger NLRP3 activation, promoting the release of proinflammatory cytokines IL-1β and IL-18 [44, 45]. Additionally, studies in mouse macrophages reveal the anti-inflammatory effect of selenium through upregulating the synthesis of PGD_2_-derived cyclopentenone prostaglandins (CyPG), Δ^12^-PGJ_2_, and 15-deoxy-∆^12,14^-prostaglandin J_2_ (15d-PGJ_2_), which also serve as endogenous agonists for the nuclear receptor, peroxisome proliferator–activated receptor gamma (PPARγ) [16]. Activation of PPARγ inhibits NFκB activation, while upregulating the expression of anti-inflammatory genes such as Arg1 and IL-10 [46], as well as inhibiting neutrophil migration and promote neutrophil apoptosis [47]. Thus, it appears that by controlling the ROS accumulation and metabolism of bioactive lipid mediators, selenium likely regulates the activation of proinflammatory signaling pathways and cytokine production in neutrophils.

## Selenium and Neutrophil Apoptosis

A few studies have demonstrated the association of dietary selenium with neutrophil apoptosis. Cattle maintained on an inorganic selenium diet showed increased neutrophil apoptosis compared to selenium-deficient animals [48]. In carp, selenium supplementation rescued tetrabromobisphenol A–induced neutrophil death [39]. In addition, ex vivo Trsp^fl/fl^S100A8^Cre^ neutrophils showed reduced neutrophil apoptosis when cultured with *C. rodentium* [26]. Neutrophil apoptosis is an important event in the resolution of an inflammatory response, through its subsequent clearance by macrophages via efferocytosis [26]. Along with the reduced neutrophil apoptosis, we observed a significant reduction in macrophage efferocytosis when cultured with Trsp^fl/fl^S100A8^Cre^ neutrophils, contributing to increased inflammation. At the whole organismal level, such a process led to reduced intestinal barrier integrity during bacterial infection leading to unresolved intestinal inflammation [26]. ROS play a dual role in regulating neutrophil apoptosis, by promoting neutrophil apoptosis through inducing caspase-8 cleavage while downregulating neutrophil apoptosis via upregulating the expression of NFκB-targeted anti-apoptosis factors such as BCL2 and XIAP [8]. Selenium might mediate neutrophil apoptosis through ROS modulation of NFκB activation to aid in the subsequent resolution of inflammation.

## Selenium and Neutrophil Development

Selenium supplementation is associated with neutrophil maturation. A case study of a patient with chronic neutropenia showed reduced serum selenium, while daily selenium supplementation increased neutrophil cell count in the peripheral blood, and neutrophil cell number returned to the original level after ceasing selenium supplementation [49]. In cattle, long-term selenium supplementation led to neutropenia [50]. Furthermore, analysis of bone marrow cells in calves showed an increased population of granulocyte precursors while matured neutrophils were reduced, shedding light on the involvement of selenium and selenoprotein in mediating neutrophil development [50].

## Selenoproteins in Neutrophils

### Glutathione Peroxidases

**Fig. 1:**
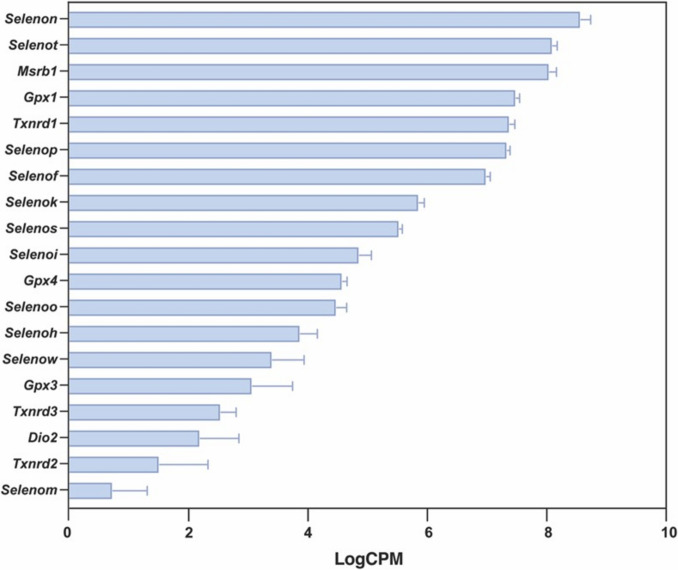
Expression of selenoproteins in murine bone marrow–derived neutrophils. RNAseq analysis was performed on naïve bone marrow isolated-Ly6G^+^ cells, and selenoprotein expression was extracted and plotted (*n* = 4). Nineteen Out of the 24 selenoprotein mRNA transcripts were detected in neutrophils without stimulation. *Selenon*, *Selenot*, *Msrb1*, *Gpx1*, *Txnrd1*, *Selenop*, and *Selenof* showed relatively higher expression, at least 2–6 folds than the rest of the selenoproteins. Data used for this analysis was extracted from repository BioProject PRJNA1304000

The GPX family is among the earliest identified Sec-containing proteins involved in buffering the cellular oxidative stress and consequently regulating redox-related signaling pathways [51]. Using glutathione as a co-substrate, GPXs reduce hydrogen peroxide through oxidizing glutathione to glutathione disulfide [51]. In eukaryotes, the GPX family consists of eight members of which only GPX1, GPX2, GPX3, GPX4, and GPX6 (in humans) contain Sec, and the expression of these proteins is tissue specific [51]. Gpx1 expression is regulated by PU.1, a transcription factor that is critical for neutrophil maturation, suggesting the plausible role for GPX1 in neutrophil development [52]. *Gpx1*, *Gpx3*, and *Gpx4* expression in mouse neutrophils was detected by RNAseq (Fig. 1). Interestingly, protein expression of Gpx2 and Gpx4 increased following LPS stimulation of neutrophils, while Gpx1 expression gradually reduced, suggesting temporal regulation of GPXs during inflammation [22]. GPX1 was the first identified selenium-containing protein, whose expression was determined to be highly dependent on dietary selenium intake [51]. Due to its ability to regulate endogenous levels of hydrogen peroxide, GPX1 is thought to regulate redox-related signaling pathways that impinge on regulating neutrophil function. Indeed, *Gpx1*^*-*/-^ mice showed an alteration in neutrophil migration efficiency during inflammation. *Gpx1*^*-*/-^ mice showed increased neutrophil accumulation in bronchoalveolar lavage fluid (BALF) upon exposure to cigarette smoke, and treatment with ebselen (a GPX1 mimic) inhibited neutrophil abundance in BALF [53]. However, intranasal stimulation with LPS reduced neutrophil accumulation in the BALF in *Gpx1*^*-*/-^ mice [54]. While the underlying reasons for this discrepancy are unclear, given the modulation of cell adhesion molecule expression in the epithelial cells by GPX1 [55], neutrophil-specific *Gpx1*^*-*/-^ mice will need to be generated to evaluate the specific role of GPX1 in neutrophil functions, particularly neutrophil migration.

GPX2 is expressed highly in the gastrointestinal tract, predominately in the basal cells [56]. Due to the overlapping substrate preference between GPX1 and GPX2, there is some compensation reported [56]. Similar to the potential role of GPX1 in regulating neutrophil recruitment during inflammation, lack of Gpx2 also resulted in increased neutrophil accumulation in the lung upon hypoxia-induced lung injury [57]. GPX2 is a downstream target of Nrf2 [57], which has been found to regulate the functions of neutrophils in response to redox stress [58]. Therefore, GPX2 may be a downstream effector of Nrf2 during neutrophil activation. Of note, protein expression of Gpx2 was detected in neutrophils that gradually increased following LPS stimulation, in contrast to the reduction in Gpx1 expression [22], suggesting a temporal translational control in Gpx2 expression.

GPX3 has structural and functional similarities to GPX1 and is mostly released into extracellular space [51]. Expression of GPX3 has been found to be negatively correlated with various cancers, and elevated plasma GPX3 has been found in children with inflammatory bowel disease (IBD) [59]. Neutrophils from RA patients showed increased expression of *GPX3* with increased ROS accumulation [60]. In chicken-derived *Gpx3*^*-*/-^ neutrophils, protein kinase C (PKC) expression was downregulated, and PMA-induced NETosis was inhibited [61]. Young et al. identified a microRNA miR-1696 that targeted *Gpx3*, and treatment with miR-1696 mimic resulted in the inhibition of MAPK signaling pathway, therefore downregulating NETs formation in neutrophils [61]. Interestingly, the same group observed selenium deficiency also resulted in increased expression of mirR-1696 expression in the small intestine, associated with reduced expression of Gpx3 that was associated with increased injury and inflammation in the chicken small intestine [62]. Furthermore, Gpx3 depletion and mirR-1696 expression in neutrophils increased ROS accumulation [61]. These results suggest GPX3 plays a role in regulating neutrophil redox status and inflammatory functions.

GPX4 has distinct functions compared to the other Sec-containing GPXs, where it not only reduces hydrogen peroxide but also lipid hydroperoxides [59]. GPX4 is an endogenous inhibitor of ferroptosis, an iron-dependent lipid peroxidation–induced cell death [17]. GPX4 expression was found to be significantly downregulated in neutrophils in lupus patients. IFNγ and autoantigen stimulated nuclear translocation of the transcriptional repressor cAMP response element modulator α (CREMα), which bound to the *GPX4* promoter and downregulated its expression in neutrophils, resulting in increased neutrophil ferroptosis leading to the pathology of lupus [17]. Additionally, ex vivo LPS stimulation increased Gpx4 expression in neutrophils, which correlated with increased lipid hydroperoxide accumulation, suggesting GPX4 involvement in neutrophil inflammation [22]. Of note, studies have shown that neutrophils, despite being enriched in lipid peroxides during activation, are resistant to ferroptosis [63]. The fact that the Trsp^fl/fl^S100a8^Cre^ neutrophils did not undergo increased cell death suggests the involvement of an alternative defense mechanism, such as ferroptosis suppressor protein 1 (FSP1) or heat shock protein beta 1 (HSPB1)–mediated ferroptosis suppression in counteracting lipid oxidative stress during neutrophil activation [22, 63].

### Thioredoxin Reductases

Thioredoxin reductases (TXNRDs) are Sec-containing disulfide oxidoreductases involved in thioredoxin-dependent redox homeostasis via reducing disulfide bonds in substrates, including thioredoxin and other non-protein substrates [64]. There are three TXNRD proteins: TXNRD1 is located in the cytoplasm; TXNRD2 and TXNRD3 are in mitochondria [64]. Neutrophils have relatively low numbers of mitochondria [1], which likely explains the low expression of *Txnrd2* and *Txnrd3*, whereas the expression of *Txnrd1* is among the top five selenoproteins in neutrophils (Fig. 1). Compared to other selenium-sensitive selenoproteins such as GPX1 and SELENOW, TXNRDs are generally less sensitive to selenium levels [65]. However, under severe selenium deficiency, Sec residue can be replaced by cysteine, which significantly reduces the enzymatic activity [66]. TXNRD1 expression in neutrophils has been linked to methylmercury-induced autism neuroinflammation, where children with autism have increased TXNRD1 expression while reduced thioredoxin expression and neutrophils from autism children are more susceptible to methylmercury-induced redox stress [67]. Muri et al. [68] found that Txnrd1 was required for T cell development but not myeloid lineage development during normal homeostasis in mice. However, depletion of Txnrd1 significantly downregulated the myeloid cell population in bone marrow and peripheral blood under redox stress [68]. Furthermore, LPS challenge reduced the neutrophil population in the bone marrow and peripheral blood in *Txnrd1*^*-*/-^ mice, suggesting its participation in emergency granulopoieses during inflammation [68]. Auranofin, a drug for treating RA, initially received great attention for its ability to modulate neutrophil function. Thought to be a PKC inhibitor [69], auranofin was later found to also be an inhibitor of TXNRD1 and TXNRD2, acting through direct binding to the active site selenocysteine [70, 71]. Treatment of auranofin at concentrations relevant to RA patient plasma auranofin (< 1 M) inhibited neutrophil apoptosis while increasing ROS production and lysosome release [72, 73], as well as altered lipid metabolism and expression of adhesion molecules in neutrophils [72, 74]. In addition, treatment with auranofin significantly inhibited neutrophil adhesion and chemotaxis against LTB_4_, suggesting a potential involvement of TXNRD1 in regulating neutrophil chemotaxis [74]. Indeed, through the interaction with focal adhesion kinase (FAK), Txnrd1 restored nitric oxide–induced S-nitrosylation on β2 Integrin during hyperoxia, which promoted neutrophil adhesion upon fMLP stimulation [75]. In CGD patients, TXNRD1 regulated TNFα expression in neutrophils through the nuclear translocation of thioredoxin, which further promoted nuclear translocation of NFκB and the expression of its downstream proinflammatory target genes [76]. Interestingly, thioredoxin-interacting protein (TXNIP) was found to regulate the activation of the inflammasome, where the oxidation of thioredoxin led to its dissociation from TXNIP, enabling the binding of TXNIP with NLRP3 to promote NLRP3 activation and IL-1β secretion [44, 77].

### Other Selenoproteins

In addition to the above, a few other selenoproteins expressed in neutrophils are reviewed below for their direct or indirect role in neutrophil functions. SELENOK was found to promote neutrophil migration toward CXCL1, and in a whole-body *Selenok*^*-*/-^ mouse, neutrophil migration during peritonitis was downregulated [78]. Neutrophils have the highest expression and activity of MSRB1 when compared to other immune cells, which is responsible for reversing oxidized methionine by reducing methionine sulfoxide to methionine [79]. Neutrophil MPO contributes to the methionine sulfoxide formation, which is required for intracellular killing of bacteria, and methionine sulfoxide reductases expression in bacteria prevents the bactericidal activity of MPO-derived hypochlorous acid [80]. MSRB1 expression might contribute to protecting neutrophils from MPO-induced oxidative stress in a similar manner. Interestingly, MSRB1 expression is found to be low in neutrophils isolated from Alzheimer patients with low MSRB1 expression in the brain, suggesting its potential as a biomarker for Alzheimer’s disease [81]. Selenof is found to be regulated by apoptotic cell-derived hydrogen sulfide that promotes its binding to STAT1 to regulate TH17 cell development [82]. Interestingly, hydrogen sulfide was found to affect neutrophil NETosis and migration [83, 84]. Additionally, NETs and neutrophil-derived cytokines were also found to regulate TH17 cell differentiation [85]. As neutrophils are able to efferocytose apoptotic cells during inflammation [86], SELENOF might contribute to the regulation of neutrophil function and its communication with neighboring cells in the immune microenvironment. Mature neutrophils express a relatively low level of *Selenoh* and *Selenow* (Fig. 1). However, transcriptomic analysis of neutrophil maturation in zebrafish showed a gradual reduction of *Selenoh* expression, while an increase in *Selenow* expression as neutrophils mature was seen, suggesting the potential involvement of SELENOH and SELENOW in neutrophil development [87]. RNAseq analysis indicated *Selenon* was the most highly expressed selenoprotein in murine neutrophils (Fig. 1), even though its expression in neutrophils is among the lowest compared to the other immune cells [88]. As a key regulator in calcium homeostasis, SELENON is found to play a role in regulating muscle regeneration [15]; however, its involvement in immune cells is unclear. Interestingly, *SELENON* expression is associated with the disease outcome of multiple cancers, where it correlated with the infiltration and activation of multiple immune cells, including neutrophils [89]. In addition, *SELENON* expression in glioma correlated with the NETs formation pathway [89]. Considering the critical involvement of calcium signaling in multiple neutrophil functions, it is likely that SELENON might be involved in regulating neutrophil activation.

## Conclusions

**Fig. 2:**
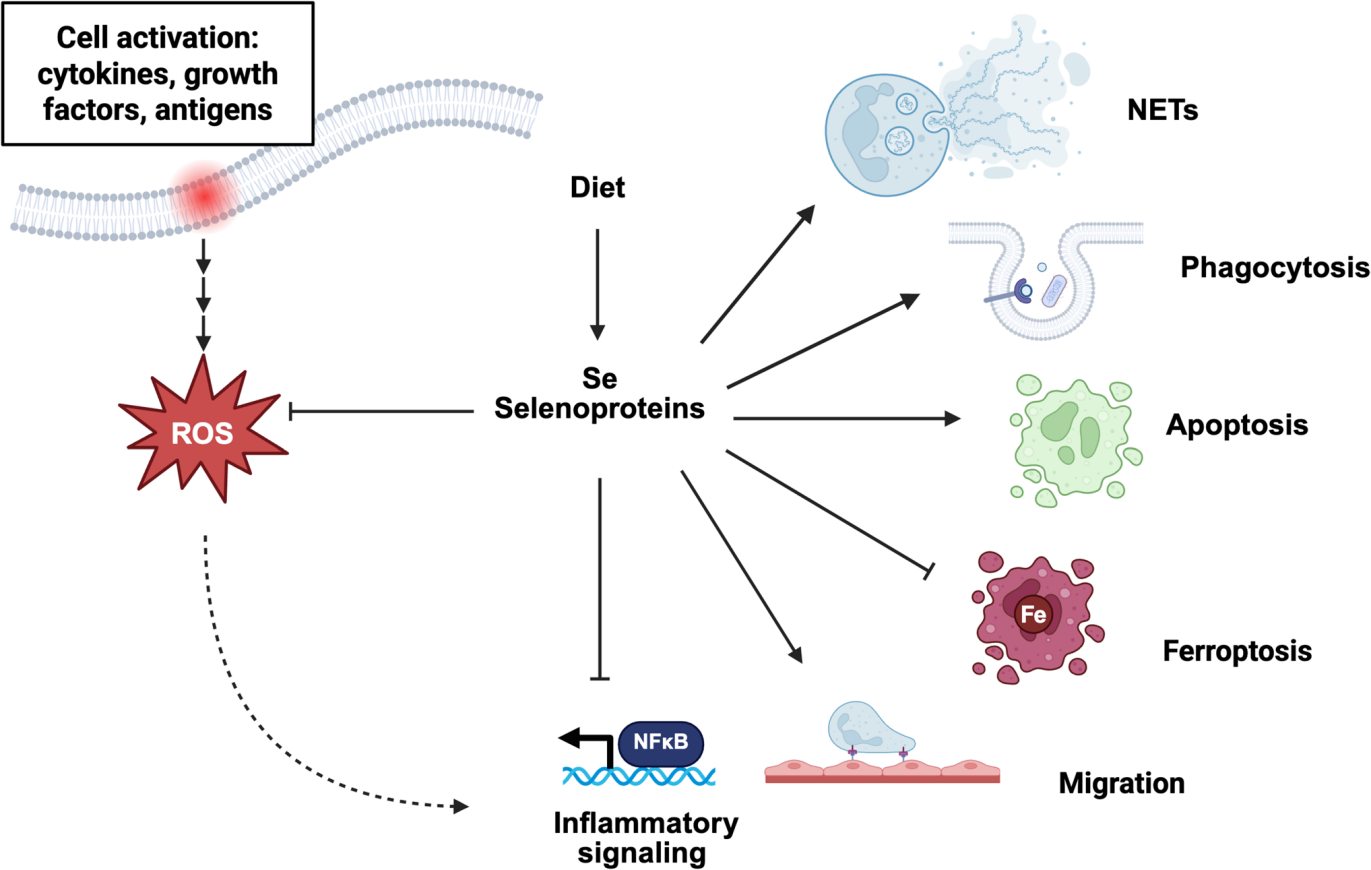
Selenium and selenoproteins regulate neutrophil functions. Activation by cytokines, growth factors, or various inflammatory stimuli triggers downstream signaling leading to ROS production in neutrophils to modulate their functions. Selenium and several selenoproteins regulate redox-dependent pathways that influence neutrophil functions, including ROS production, migration, apoptosis, ferroptosis, NET formation, and activation of inflammatory signaling

Neutrophils are subjected to redox stress during inflammation, either by endogenous ROS or by ROS from surrounding immune cells and host tissue. The involvement of ROS in various functions of neutrophils, and the ability of selenium and selenoproteins to regulate ROS production and accumulation suggest a strong possibility of their role in neutrophil-mediated inflammation and cellular function. Indeed, modulation in neutrophil inflammatory functions and cell death has been observed, shedding light on the role of selenoproteins in neutrophils (Fig. 2, Table 1). However, many of them are based on studies involving manipulations in the diet or genetic mutations at the organismal level, which can lead to non-specific effects, as neutrophil function is largely regulated by interacting cells and other factors (e.g., hormones, cytokines, chemokines) [21], making it challenging to accurately assess the role of selenoproteins in neutrophil functions. Future studies will require the development of neutrophil-targeted deletion of specific selenoproteins to elucidate the mechanism of selenoprotein regulation in neutrophils under physiological and pathological conditions.

**Table 1:** Summary of studies focused on the role of selenoproteins in the regulation of neutrophil functions

Gene		Species	Citation
GPX1	*Gpx1*^*-*/-^ affected neutrophil recruitment	Mouse	[53, 54]
GPX2	*Gpx2*^*-*/-^ increased neutrophil accumulation	Mouse	[57]
GPX3	Promoted NETosis via MAPK activation	Chicken	[61, 62]
	Negatively regulated ROS accumulation in neutrophil	Chicken	[61]
GXP4	Inhibited neutrophil ferroptosis	Human	[17]
	Expression in neutrophils increased with LPS stimulation	Mouse	[22]
TXNRD1	Expression increased in neutrophils from autistic children	Human	[67]
	Whole body KO affected neutrophil abundance in bone marrow and peripheral blood under LPS challenging	Mouse	[68]
	Inhibition of Txnrd1 by auranofin inhibited neutrophil apoptosis	Human	[72]
	Inhibition of Txnrd1 by auranofin increased neutrophil ROS production and lysosome release	Human	[72, 73]
	Inhibition of Txnrd1 by Auranofin altered lipid metabolism and adhesion molecules expression in neutrophils	Human	[72, 74]
	Positively regulated neutrophils chemotaxis and adhesion by reversing β2 Integrin S-nitrosylation	Mouse	[75]
	Inhibition of Txnrd1 inhibited NFκB nuclear translocation and TNFα expression in neutrophils from CGD patients	Human	[76]
SELENOK	Promoted neutrophil migration	Mouse	[78]
	*Selenok*^*-*/-^ inhibited neutrophil recruitment	Mouse	[78]
MSRB1	Low MSRB1 expression observed in neutrophils from patients with Alzheimer’s disease showing reduced MSRB1 expression in brain	Human	[81]
SELENOH	mRNA expression reduced during neutrophil maturation	Zebrafish	[87]
SELENOW	mRNA expression increased during neutrophil maturation	Zebrafish	[87]

## Data Availability

No datasets were generated or analysed during the current study.

## Abbreviations

*ALOX12*: Arachidonate 12-lipoxygenase
*COX*: Cyclooxygenase
*fMLP*: N-formyl-methionyl-leucyl-phenylalanine
*GPX*: Glutathione peroxidase
*KO*: Knockout
*LOX*: Lipoxygenase
*LTB*_*4*_: Leukotriene B4
*MPO*: Myeloperoxidase
*MSRB1*: Methionine sulfoxide reductase B1
*NE*: Neutrophil elastase
*NETs*: Neutrophil extracellular traps
*NOX*: NADPH oxidases
*PRRs*: Pattern recognition receptors
*PG*: Prostaglandins
*RA*: Rheumatoid arthritis
*ROS*: Reactive oxygen species
*Se*: Selenium
*Sec*: Selenocysteine
*SeNP*: Selenium-containing nanoparticle
*TRSP*: TRNA-SeC (anticodon TCA) 1-1 (TRU-TCA1-1)
*TXNRD*: Thioredoxin reductase
